# The Channel-Kinase TRPM7 as Novel Regulator of Immune System Homeostasis

**DOI:** 10.3390/cells7080109

**Published:** 2018-08-17

**Authors:** Wiebke Nadolni, Susanna Zierler

**Affiliations:** Walther Straub Institute of Pharmacology and Toxicology, Faculty of Medicine, LMU Munich, Goethestr. 33, 80336 Munich, Germany; wiebke.nadolni@lrz.uni-muenchen.de

**Keywords:** TRPM7, kinase, inflammation, lymphocytes, calcium signalling, SMAD, TH17, hypersensitivity, regulatory T cells, thrombosis, graft versus host disease

## Abstract

The enzyme-coupled transient receptor potential channel subfamily M member 7, TRPM7, has been associated with immunity and immune cell signalling. Here, we review the role of this remarkable signalling protein in lymphocyte proliferation, differentiation, activation and survival. We also discuss its role in mast cell, neutrophil and macrophage function and highlight the potential of TRPM7 to regulate immune system homeostasis. Further, we shed light on how the cellular signalling cascades involving TRPM7 channel and/or kinase activity culminate in pathologies as diverse as allergic hypersensitivity, arterial thrombosis and graft versus host disease (G_V_HD), stressing the need for TRPM7 specific pharmacological modulators.

## 1. Introduction

The melastatin-like TRPM7 channel conducts divalent cations, specifically calcium (Ca^2+^), magnesium (Mg^2+^) and zinc (Zn^2+^) [1,2,3]. It has been implicated in cellular and systemic Mg^2+^ homeostasis [4,5,6], Zn^2+^-mediated toxicity [7,8] and intracellular Ca^2+^ signalling [9,10,11,12]. The TRPM7 channel is considered to be constitutively active and its activity to be negatively regulated by intracellular cations (Mg^2+^, Ba^2+^, Sr^2+^, Zn^2+^, Mn^2+^), Mg-ATP, polyamines, chloride (Cl^−^) and bromide (Br^−^) concentrations, low intracellular pH and hydrolysis of the acidic phospholipid phosphatidylinositol 4,5-bisphosphate (PIP_2_) [13,14,15,16]. Resting free cytosolic Mg^2+^ (0.5–1 mM [Mg^2+^]_c_) and Mg-ATP (3–9 mM) concentrations [17] seem to be sufficient to block native TRPM7 channel activity [4,18,19,20]. TRPM7’s unique enzyme encodes a serine-threonine kinase closely related to eukaryotic elongation factor-2 kinase [21], phosphorylating mainly within α-helical loops [22]. A few in vitro TRPM7 kinase substrates have been identified early on, including annexin A1 [23,24], myosin II heavy chain [25] and PLCγ2 [26]. Only recently, with the development of novel mouse models, the first native kinase substrate, SMAD2, was discovered, paving the way for more to follow [11,20,27]. 

**Figure 1:**
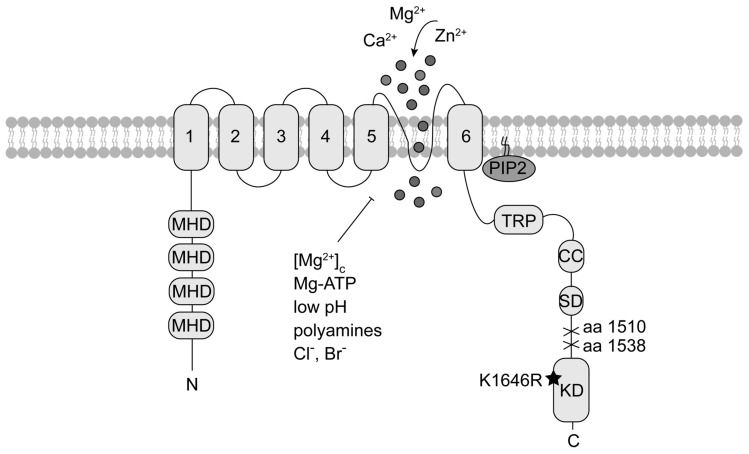
TRPM7 topology. Each TRPM7 protein consists of six transmembrane segments (1 to 6) with the channel pore located between segment 5 and 6. Within the N-terminus are melastatin homology domains (MHD), characteristic for TRPM family members. The cytoplasmic *C*-terminus contains a transient receptor potential domain (TRP), a coiled-coil domain (CC) and a kinase substrate domain (SD) upstream the atypical α-type serine/threonine protein kinase domain (KD). Mutation at the catalytic side of the kinase (K1646R) abolishes kinase activity without affecting current activity. Deletion of the KD at different amino acids (aa) results in either enhanced or reduced current activity. The black star indicates the location of the point mutation, crosses mark the kinase deletion. TRPM7 is negatively regulated by depletion of phosphatidylinositol 4,5-bisphosphate (PIP_2_), physiologic free cytosolic magnesium concentrations [Mg^2+^]_c_, Mg-ATP, polyamines, low pH and chloride (Cl^−^) and bromide (Br^−^) concentrations.

Genetic disruption of TRPM7 in mice (*Trpm7^−/−^*) is embryonic lethal [4,28]. Deletion of the exons encoding the TRPM7 kinase domain only (*Trpm7^Δk/Δk^*) also leads to early embryonic lethality [4]. However, the latter phenotype could be attributed to a reduction in channel activity in this mutant, particularly, as mice bearing a single point mutation at the active site of the kinase (K1646R, *Trpm7^R/R^*), thus inactivating its catalytic activity, are viable and display no obvious phenotype [29,30]. Heterozygous *Trpm7^+/Δk^* mice are also viable but develop hypomagnesaemia upon Mg^2+^ restriction [4]. Deletion of the TRPM7 kinase domain at amino acid (aa) 1538 yields in reduced current amplitudes, while caspase-induced deletion at aa 1510 results in enhanced TRPM7 currents [4,31]. Inactivation of kinase activity via the K1646R mutation (*Trpm7^R/R^*), though, does not affect current development [29,30,32] (Figure 1). However, it was reported to show increased basal current activity right after break-in in macrophages [29]. Recently, it was shown that the Mg^2+^-sensitivity of the TRPM7 channel is reduced almost two-fold in kinase-deficient *Trpm7^R/R^* mouse mast cells. The tested Mg^2+^ concentrations, however, suggest that this effect will not influence TRPM7 currents in intact cells with [Mg^2+^]_c_ close to 1 mM [20]. Accordingly, how TRPM7 channel and kinase activities affect each other is still incompletely understood. It is thought that they are interdependent in that Mg^2+^ enters through the channel pore and the kinase domain requires Mg^2+^ ions to function [3,22], while the kinase domain rather than the catalytic activity are crucial for channel function [4,29,30,31].

TRPM7 kinase requires Mn^2+^ or Mg^2+^ for its activity and uses mainly Mg-ATP for phosphorylation [22]. Massive auto-phosphorylation of TRPM7 increases kinase activity and substrate recognition [33,34,35,36]. Considering the ubiquitous expression of TRPM7, it is not surprising that the protein has a fundamental and non-redundant role in cellular physiology [1,37]. It is involved in processes as diverse as proliferation, growth, migration, apoptosis, differentiation and exocytosis [38]. Tissue-specific deletion of TRPM7 in thymocytes or macrophages, as well as inactivation of the kinase activity (*Trpm7^R/R^*) in mice, highlight the importance of this unique signalling protein for an operating immune system that is still kept in check [20,28,39].

## 2. The Channel-Kinase TRPM7 in Immune Cell Signalling

### 2.1. TRPM7 Kinase Regulates Mast Cell Reactivity

Mast cells are associated with the progression of different pathologies such as immediate and delayed hypersensitivity reactions, arthritis, atherosclerosis, heart failure, as well as neuroinflammatory diseases [40,41,42]. Upon stimulation, mast cells release granules, filled with inflammatory mediators such as histamine, proteases, cytokines and growth factors [43,44]. Mast cells are present in all organs in close proximity to blood vessels, neurons and lymphatic vessels, thus disseminating local inflammatory signals [40]. While classically, mast cells are activated via crosslinking of the IgE receptor (F_c_εRI) upon antigen binding [45], their activation can be triggered by many other stimuli including toll-like receptor (TLR) ligands, complement and neuropeptides. Receptor stimulation in mast cells leads to a network of stimulatory and inhibitory signals [44] encoded via intricate Ca^2+^ signalling events [46,47]. Store operated Ca^2+^ entry is essential for mast cell activation in vitro and in vivo [48,49]. Recently, TRPM7 has been implicated in receptor-induced Ca^2+^ release as well as store-operated Ca^2+^ entry [9,12]. In primary human lung mast cells (HLMCs) and in the human mast cell lines, LAD2 and HMC-1, TRPM7 expression and function was shown to be essential for cell survival. Using adenoviral-mediated knock-down of TRPM7 in HLMC or HMC-1 authors observed enhanced cell death, which was not rescued by extracellular Mg^2+^ supplementation [50]. First indications that TRPM7 is involved in degranulation processes and release of cytokines in rat bone marrow-derived mast cells (BMMCs) were gained in 2014. TRPM7 mRNA expression levels were significantly higher in asthmatic rat BMMCs than in controls. Genetic or pharmacological inhibition of TRPM7 significantly decreased β-hexosaminidase activity and secretion of histamine as well as the release of the pro-inflammatory cytokines IL-6, IL-13 and TNF-α in the asthmatic group compared to the control group. Authors concluded that inhibition of TRPM7 currents reduces mast cell degranulation and cytokine release [51]. However, functional TRPM7 channel or kinase activities were not shown. The pharmacological antagonist used to inhibit TRPM7, 2-Aminoethoxydiphenyl Borate (2-APB), supposedly only blocks channel and not kinase activity but has various off-target effects. Importantly, at the applied concentrations (100–200 µM) 2-APB blocks store operated Ca^2+^ entry (SOCE) via inhibition of Ca^2+^ release activated calcium (CRAC) channels [52]. Thus, it is possible that observed 2-APB effects were in fact not mediated by TRPM7 but SOCE. Authors, however, confirm the reduction in β-hexaminidase activity as well as in histamine and cytokine release, using a lentiviral siRNA-mediated knock-down of TRPM7 in primary BMMCs [51]. Nonetheless, the question remained, whether kinase and/or channel activity were responsible for the observed phenotype. As kinase-deficient *Trpm7^R/R^* mutant mouse mast cells showed normal current amplitudes but no kinase activity, this model allowed the independent study of TRPM7 channel versus kinase moieties in mast cells [32]. Utilizing the two TRPM7 kinase mutant mouse models (*Trpm7^+/Δk^*, *Trpm7^R/R^*), it was shown that the kinase regulated G protein-coupled receptor-activated histamine release, independently of channel activity. TRPM7 kinase activity, moreover, regulated Ca^2+^-sensitivity of G protein-triggered mast cell degranulation. TRPM7 kinase-deficiency resulted in suppressed IgE-dependent exocytosis and slower cellular degranulation rates. Besides, extracellular Mg^2+^ was necessary to guarantee regulated IgE-induced exocytosis. Authors concluded that the TRPM7 kinase activity controls murine mast cell degranulation and histamine release independently of TRPM7 channel function [32]. Thus, TRPM7 might inflict its immune-modulatory role on mast cells via its kinase domain. 

### 2.2. TRPM7 in Neutrophil Migration

Neutrophils, the most abundant leukocytes in the blood, are one of the key players of the innate immune system, contributing to the clearance of acute inflammation and bacterial infection [53]. During acute inflammation neutrophils are one of the first responding cells. The signalling cascades triggered after neutrophil activation start with migration of neutrophils towards the inflammatory site. The cascade ends with elimination of pathogens via secretion of chemokines, which attract other leukocytes, phagocytosis to maintain tissue homeostasis and degranulation and release of neutrophil extracellular traps (NETs) to prevent the spread of the infection. It is well established that Ca^2+^ signalling is pivotal for the recruitment cascade and activation of neutrophils, highlighting the importance of ion channels for neutrophil function [54,55]. TRPM7 channel activity has been implicated as a regulator of cell migration by facilitating Ca^2+^ oscillations [56,57,58]. Using a human neutrophil cell line, it was shown that TRPM7 is recruited into lipid rafts in a CD147-dependent manner. Knock-down of CD147, a glycoprotein required for neutrophil recruitment and chemotaxis, caused significant decrease in lipid raft localization of TRPM7. Thus, TRPM7 was suggested to be involved in the CD147-triggered Ca^2+^-induced chemotaxis, adhesion and invasiveness of human neutrophils [59]. However, Wang and colleagues did not show functional TRPM7 ion channel activity and one needs to keep in mind that neutrophil-like cell lines are controversially discussed regarding their comparability to primary neutrophils [53]. During acute lung injury (ALI), the permeability of the alveolar-capillary membrane is increased, which in turn can lead to migration of neutrophils [60]. A septic rat model treated with salvianolic B showed that sepsis-induced ALI was reduced due to decreased levels of TRMP6 and TRPM7 mRNA in lung tissue, potentially linking TRPM7 to neutrophil migration and infiltration [61]. As patients with inherited neutrophil deficiencies suffer from severe infections that are often fatal, underscoring the importance of this cell type in immune defence, it is critical to gain a better understanding of the role of TRPM7 channel and kinase activities in the signalling cascades triggering neutrophil migration [53].

### 2.3. TRPM7 Guides Macrophage Activation and Polarization

Blood monocytes and tissue macrophages are major components of innate immunity, strategically positioned throughout the body tissues to orchestrate inflammatory processes. Similar to neutrophils, they maintain tissue homeostasis via phagocytosis of dead cells, debris or potentially harmful pathogens. As antigen presenting cells they are able to activate and coordinate the adaptive immune system [62,63]. Various TRP proteins have been associated with macrophage-mediated inflammatory responses [64]. TRPM7 has also been linked to the activation and proliferation of monocytes and macrophages [29,65,66,67,68]. However, controversies on the role of TRPM7 for macrophage activation in response to LPS remain [39,68]. On the one hand, TRPM7 channel activity was suggested to be essential for macrophage proliferation and polarization into the alternate or anti-inflammatory M2-subtype [68]. LPS and co-stimulatory cytokines IFN-γ (pro-inflammatory M1-type) or IL-4 and IL-13 (anti-inflammatory M2-type) trigger the polarization of macrophages [69,70]. Interestingly, the activity of TRPM7 increased significantly in response to stimulation with IL-4. The TRPM7 inhibitor NS8593 [71] blocked IL-4 and M-CSF induced proliferation and reduced the inhibitory effect of IL-4 or M-CSF on the LPS-induced expression of the pro-inflammatory cytokine TNF-α, thus, counteracting the differentiation into the M2 subtype [68]. On the other hand, more recently, TRPM7 channel activity has been implicated in macrophage activation in response to LPS and LPS-induced peritonitis [39]. In TRPM7-deficient macrophages (*Trpm7^fl/fl^*(*LysM Cre*)) IL-1β secretion was significantly reduced and also the gene expression upon LPS stimulation was altered, indicating a key function of TRPM7 in the activation process of macrophages. In addition, it was found that TRPM7 is pivotal for the endocytosis of LPS-TLR4-CD14 signalling complexes with TRPM7-deficient macrophages showing significantly reduced internalization of TLR4 and CD14. Schappe et al. demonstrated that these defects upon LPS stimulation were due to diminished TRPM7-mediated Ca^2+^ influx. They speculate that TRPM7 not only controls TLR4 internalization but also regulates downstream IRF3 and NFκB signalling by mediating cytosolic Ca^2+^ elevations. Moreover, in a LPS-dependent model of peritonitis, *Trpm7^fl/fl^* (LysM Cre) mice had decreased serum cytokine levels after LPS treatment, preventing pathological inflammation. Specifically, the expression levels of *Tnfa* and *Il1b* were significantly reduced, resulting in a diminished recruitment of immune cells into the peritoneum. Thus, *Trpm7^fl/fl^* (LysM Cre) mice were protected from the development of LPS-induced peritonitis. Consequently, it was suggested that TRPM7 channel blockade could be beneficial for the treatment of chronic infections or septic shock [39]. The difference in the macrophage response to LPS might depend on different protocols used. However, to date there is no consensus, whether LPS induces Ca^2+^ elevations resulting in macrophage activation. Several studies have found no changes in cytosolic Ca^2+^ concentrations in response to LPS treatment in macrophages [72,73].

The role of TRPM7 kinase activity in macrophage or dendritic cell function is far less understood. TRPM7 kinase-deficient mice (*Trpm7^R/R^*) show no defects in percentages of macrophages [29]. Also, *Trpm7^R/R^* CD11c^+^ dendritic cells develop normally and display regular major histocompatibility complex II (MHCII) and integrin expression [20].

### 2.4. TRPM7 Affects Lymphocyte Functions

Lymphocytes forming the adaptive or acquired immune response are activated and regulated by cells of the innate immune system, that is, macrophages and provide immunologic memory [74]. Antigen specific lymphocytes respond to pathogens with activation induced proliferation and clonal expansion. This temporal proliferative burst is terminated with a return to cell quiescence and eventual cell death. The autonomous timing of proliferation ensures an appropriate response magnitude whilst preventing uncontrolled expansion. Thus, a detailed understanding of the regulatory principles governing lymphocyte activation, proliferation, differentiation and survival is essential to a cohesive picture of the immune system homeostasis [75]. 

#### 2.4.1. TRPM7 Kinase Regulates Intracellular Calcium Signals and Proliferation in Lymphocytes

**Figure 2:**
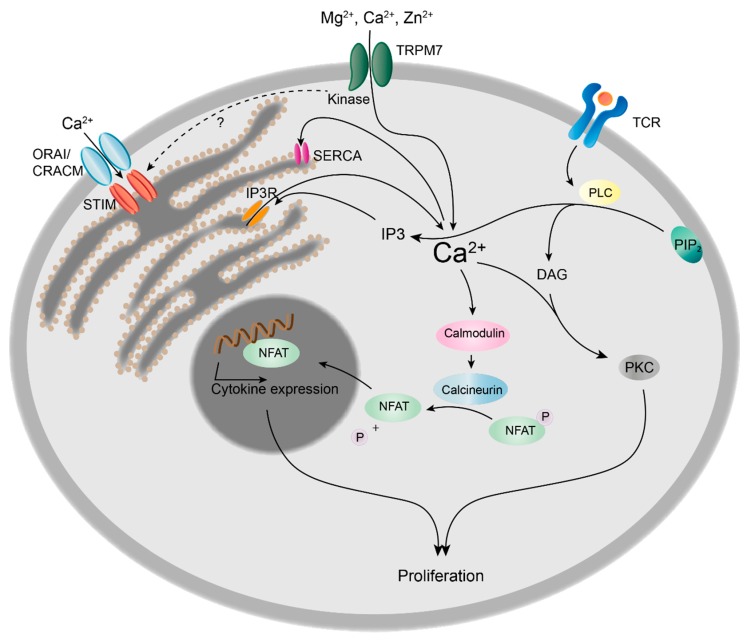
Role of TRPM7 kinase in calcium signalling and proliferation of T cells. Upon T cell receptor (TCR) binding, phospholipase C (PLC) is activated and hydrolyses phosphatidylinositol 4,5-biphosphate (PIP_2_) to inositol 1,4,5-triphosphate (IP_3_) and diacylglycerol (DAG). DAG in conjunction with Ca^2+^ activates protein kinase C (PKC), thus inducing cell proliferation. IP_3_ induces Ca^2+^ release from the endoplasmic reticulum (ER) via IP_3_ receptor (IP_3_R), followed by the translocation of the stromal interaction molecule (STIM) to the plasma membrane. STIM triggers Ca^2+^ influx from the extracellular space via ORAI/CRACM channels. Ca^2+^ is rapidly removed from the cytosol by the sarco/endoplasmic reticulum Ca^2+^-ATPase (SERCA), refilling the ER Ca^2+^ stores. The prolonged increase in cytosolic Ca^2+^ concentrations leads to the activation of calcineurin, resulting in the dephosphorylation and nuclear translocation of nuclear factor of activated T cells (NFAT) and subsequent cytokine expression, e.g., interleukin 2 (IL-2), triggering clonal expansion of T cells.

Upon T cell receptor (TCR) or B cell receptor (BCR) stimulation, phospholipase C (PLC) is activated, catalysing the hydrolysis of PIP_2_ into diacylglycerol (DAG) and inositol (1,4,5) triphosphate (IP_3_). Subsequently, IP_3_ triggers Ca^2+^-release from the endoplasmatic reticulum (ER) Ca^2+^-store via the IP_3_-receptor (IP_3_R). Upon depletion of Ca^2+^ from the ER lumen, the stromal interaction molecule (STIM) translocates to the plasma membrane and triggers SOCE via CRAC channels. This prolonged increase in intracellular Ca^2+^ concentrations is essential for the nuclear factor of activated T cells (NFAT) to translocate into the nucleus and induce transcription of genes essential for cell proliferation and clonal expansion (Figure 2) [38]. 

Recently, Romagnani et al. revealed that TRPM7 kinase-dead (*Trpm7^R/R^*) CD4^+^ T cells show slightly but significantly decreased Ca^2+^ signals upon stimulation with plate-bound anti-CD3/CD28 antibodies, whereas basal cytosolic Ca^2+^ concentrations ([Ca^2+^]_c_) were unaltered [20]. These experiments were performed using extracellular 2 mM Ca^2+^ concentrations. Similarly, Beesetty et al. showed that receptor-mediated Ca^2+^ signalling was significantly diminished in *Trpm7^R/R^* T cells using anti-CD3 crosslinking in 0.4 mM extracellular Ca^2+^ levels, and differences in 2 mM Ca^2+^ concentrations were even more pronounced. Nevertheless, basal [Ca^2+^]_c_ was also unchanged. In addition, SOCE was decreased in *Trpm7^R/R^* splenocytes upon pre-treatment with phorbol 12-myristate 13-acetate (PMA) and ionomycin, while there was no difference in ER-store Ca^2+^ content. Basal [Ca^2+^]_c_, however, were lower in PMA and ionomycin pre-activated *Trpm7^R/R^* T cells compared to wild-type (WT) [10]. These results are in line with recent studies, suggesting that TRPM7 regulates store-operated Ca^2+^ entry. Faouzi et al. linked TRPM7 channel and kinase moieties to direct involvement in SOCE. *Trpm7*-deficient chicken B lymphocytes exhibited down-regulation of SOCE, which was mainly attributed to missing kinase activity [12]. Moreover, it was shown that TRPM7 channels seem to be essential to sustain the Ca^2+^ content of intracellular stores in resting cells. Authors speculate that TRPM7 kinase may directly phosphorylate STIM2, thereby influencing Ca^2+^ entry via SOCE, yet found no effect on phosphorylation of STIM1 or STIM2 [12].The first indication that TRPM7 activity is involved in SOCE was found in 2005. Matsushita and colleagues reported that SOCE, in response to thapsigargin-induced store depletion, was increased in HEK293 cells transfected with WT TRPM7 but did not change in cells transfected with a kinase domain-deleted *TRPM7^ΔKD^* (aa 1-1599) mutant construct, compared to mock transfected controls. However, the described *TRPM7^ΔKD^* mutant displayed almost no current activity, leaving the question of the role of TRPM7 channel versus kinase moieties unanswered [76]. Also, the impact of TRPM7 kinase activity on T cell proliferation efficiency, following Ca^2+^ signalling events, remains controversial. While Romagnani et al. discovered that *Trpm7^R/R^* T cells proliferate independently of their kinase activity in response to TCR stimulation (plate-bound anti-CD3/CD28) [20], Beesetty et al. showed a reduced T cell proliferation, in response to PMA and ionomycin treatment during the first 24 hours, which was compensated for after 48 and 72 hours [10]. As the reported reduction in Ca^2+^ signalling was very small, using plate-bound anti-CD3/CD28 antibodies, it is not surprising that *Trpm7^R/R^* T cell proliferation was not altered [20]. The decrease in SOCE upon pre-treatment with PMA ionomycin in *Trpm7^R/R^* splenocytes however, was much more pronounced, resulting in reduced proliferation [10]. The combination of PMA—directly activating protein kinase C—with the calcium ionophore ionomycin, which increases [Ca^2+^]_c_, is a fast and powerful stimulus, circumventing classical receptor activation. Thus the observed alterations in proliferation depend on the experimental conditions used, suggesting that TRPM7 kinase might influence proliferation depending on the stimuli and that receptor-operated mechanisms might be compensating [10,20]. Summarizing, these studies highlight a potential role of TRPM7 kinase activity in regulating Ca^2+^ signalling and subsequent activation processes in T cells and suggest TRPM as key regulator of the temporal proliferative burst. However, how exactly TRPM7 channel and kinase activities interplay to regulate Ca^2+^ signalling and subsequent proliferation in T lymphocytes still needs further investigation. 

#### 2.4.2. TRPM7 in Cell Growth, Activation and Development of B Cells

A *Trpm7*-deficient B-lymphocyte cell line (chicken DT40 cells) exhibits a selective defect to proliferate in regular media but can do so in media supplemented with 10 mM Mg^2+^. After 24 h in regular media, TRPM7-deficient B cells accumulated in the G0/G1 phase of the cell cycle and were reduced in average cell size. Authors conclude that TRPM7-deficient cells display a defect in growth, failing to increase in size and mass. This defect was attributed to the lack of signalling downstream of phosphoinositide 3-kinase (PI3-K) with impaired mammalian target of rapamycin complex 1 (mTORC1) signalling, ribosomal S6 kinase (S6K) and Akt activation, whereas ERK phosphorylation was unaltered. Interestingly, overexpression of constitutively active AKT was not sufficient to overcome this growth defect. However, provision of a heterologous sustained PI3K signal, utilizing a constitutively active form of the catalytic subunit of PI3K, p110α, counteracted the failure of TRPM7-deficient cells to grow and proliferate in regular media. Thus TRPM7 was positioned alongside PI3K signalling as a central regulator of lymphocyte growth [77]. Moreover, TRPM7 was shown to be essential for cell-cycle progression, as *Trpm7*-deficient DT40 B cells showed an up-regulation of p27^kip^, a key cell cycle regulator which blocks the transition from G_0_ to S phase. The quiescence was reversible and rescued by Mg^2+^ supplementation or TRPM7 overexpression [78]. Utilizing the same *Trpm7*-deficient DT40 B cell line and a DT40 cell line expressing the kinase-dead mutant (K1648R), it was recently shown that TRPM7 is essential for early events of B cell activation through both kinase and channel activities. TRPM7 channel activity controlled antigen uptake and presentation to T cells [27]. Previously, TRPM7 kinase has been suggested to regulate non-muscle myosin IIA filament stability as well as actomyosin contractility by phosphorylating myosin IIA heavy chain [25], while the Mg^2+^ influx through the channel was also correlated with maintenance of myosin II-dependent cytoskeletal organization [79]. TIRF microscopy revealed that expression of TRPM7 in B cells controlled actin dynamics and slowed antigen internalization, resulting in prolonged B cell signalling. Authors conclude that TRPM7 signalling is essential for B cell affinity maturation and antibody production [27]. Moreover, recent findings indicate that TRPM7 expression is required for murine B cell development. Mice with tissue specific deletion of TRPM7 in B cells failed to generate peripheral B cells due to a developmental defect at the pro-B cell stage and increased apoptosis of B cell precursors in the bone marrow. In vitro the development of *Trpm7*-deficient B cells could be rescued via Mg^2+^-supplementation. Whereas, TRPM7 kinase-deficiency did not affect the development of B cells in the bone marrow or the percentage of peripheral B cells. Interestingly, the deletion of the entire TRPM7 protein in B cells lead to increased percentages of neutrophils, eosinophils and monocytes in the spleen of mutant mice, compared to WT which could be attributed to the primary lack of B cells [80]. Thus, TRPM7 channel and kinase activities seem unique and non-redundant for proper B cell function. 

#### 2.4.3. TRPM7 in Murine T Cell Development, Differentiation and Transcriptional Regulation

**Figure 3:**
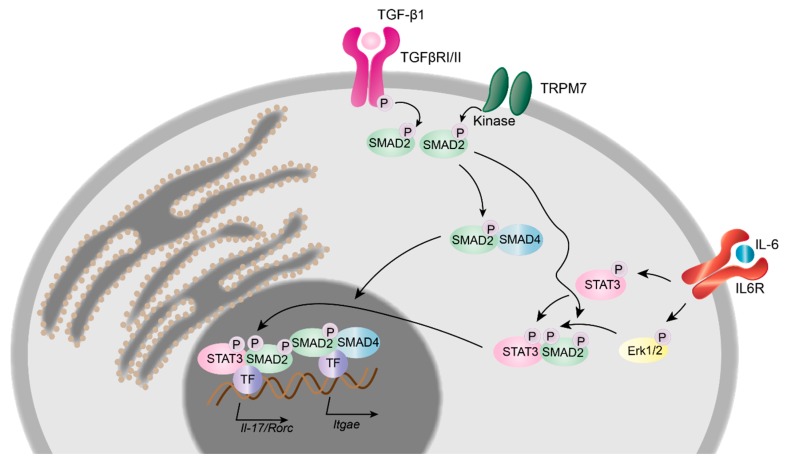
TRPM7 kinase in T cell signalling and transcriptional regulation. Upon binding of tumour growth factor β1 (TGF-β1), the TGF-β receptor complex (TGFβRI/II) initiates the phosphorylation of the c-terminal SXS-motif of SMAD2. Results gained from TRPM7 kinase deficient murine T cells suggest an additional mechanism by which the TRPM7 kinase phosphorylates SMAD2 directly, once it is anchored to the plasma membrane. Phosphorylated SMAD2 interacts with SMAD4 and promotes the transcription of *Itgae*, *Il-17* and *Rorc* genes. The interleukin 6 (IL-6) dependent STAT3 as well as Erk1/2 phosphorylation pathway is unaltered in TRPM7 kinase deficient murine T cells.

**Figure 4:**
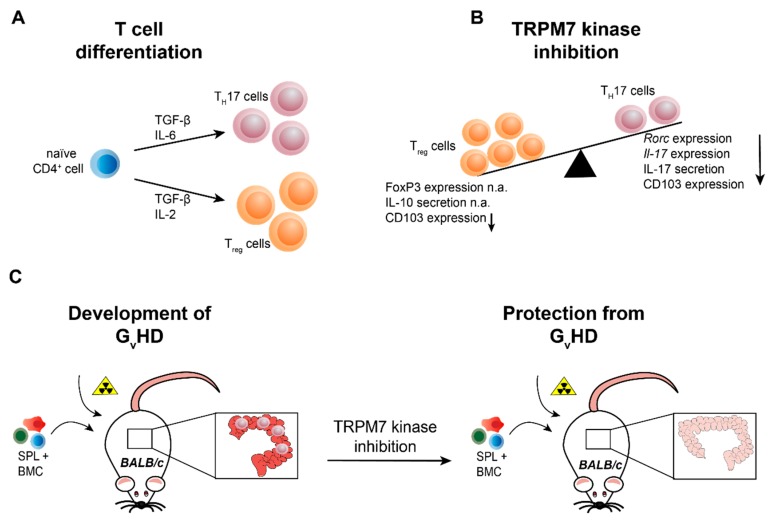
TRPM7 kinase is essential for T cell differentiation into the pro-inflammatory T_H_17 cell type and the development of graft versus host disease. (**A**) Naïve CD4^+^ T cells differentiate into pro-inflammatory T_H_17 cells in the presence of TGF-β and IL-6. For the differentiation into anti-inflammatory regulatory T cells (T_regs_), the cytokines TGF-β and IL-2 are required. (**B**) Genetic inactivation of TRPM7 kinase activity (*Trpm7^R/R^*) results in reduced T_H_17 cell differentiation, indicated via diminished *Rorc* and *Il-17* mRNA expression as well as IL-17 serum levels, while T_reg_ cell differentiation, evident via FoxP3 expression and IL-10 serum levels, is unaltered. In addition, integrin αE (CD103) expression is reduced in TRPM7 kinase deficient T cells. (**C**) Transplantation of bone marrow cells (BMC, C57BL/6) in conjunction with splenocytes (SPL, C57BL/6) triggers the development of graft versus host disease (G_V_HD) in lethally irradiated mice with different genetic background (BALB/c), manifesting in massive tissue destruction of the intestine but also lung and skin tissues. TRPM7 kinase deficient BMC and SPL transplantation does not induce inflammation in the intestine and ameliorates or even prevents disease progression, suggesting TRPM7 kinase inhibition as valid tool for the treatment of G_V_HD. n.a. (not altered).

In murine T lymphocytes TRPM7 is required for thymic development and thymopoiesis. Conditional knock-out of *Trpm7* in the T cell lineage was shown to disrupt thymopoiesis, with thymocytes remaining in the double negative (CD4^−^CD8^−^) state and resulted in altered chemokine and cytokine expression profiles [28], indicating that TRPM7 channel and/or kinase are important for T cell function. Using the homozygous kinase-dead *Trpm7^R/R^* mouse model, recently it was shown that TRPM7 kinase activity is not essential for thymopoiesis [10,20]. However, the enzymatic activity of TRPM7 is required for intra-epithelial T cell homeostasis. *Trpm7^R/R^* mice almost completely lack gut intraepithelial T lymphocytes (IELs) [20]. Intestinal IELs represent a first line of defence within the largest immune organ of our body [81]. Numerous effector T lymphocytes differentiate in the intestine, from where they migrate into the periphery [82,83]. Thus, understanding the gut immune system, harbouring ∼70% of the total lymphocytes in the human body, is of utmost importance [81,84] for regulating of immune homeostasis. Analysis of the percentage of remaining *TRPM7^R/R^* IELs revealed a significant reduction in pro-inflammatory T_H_17 cell subsets, while the percentage of anti-inflammatory T_reg_ cells was unaffected compared to WT. Consistently, the in vitro differentiation of naïve *Trpm7^R/R^* T cells into T_H_17 cells was also compromised, while the T_reg_ cell differentiation proceeded unperturbed. These findings were coherent with the robust reduction of IL-17 concentration in serum from *Trpm7^R/R^* mice [20]. As TGF-β/SMAD pathways are crucial for the polarization of CD4^+^ T cells into T_H_17 cells [85], it is likely that the TGF-β/SMAD signalling pathway is affected by TRPM7 kinase activity (Figure 3). Notably, Western Blot analysis of *Trpm7^R/R^* naïve CD4^+^ T cells treated with TGF-β1 revealed a significant reduction in SMAD2 phosphorylation, while SMAD3 phosphorylation was unaltered. Analysis of the TGF-β1-induced SMAD2 translocation was also significantly reduced in *Trpm7^R/R^* naïve CD4^+^ T cells. Thus, authors conclude that the TRPM7 kinase regulates T_H_17 cell differentiation via TGF-β/SMAD2 dependent pathways. An in vitro kinase assay using highly purified recombinant TRPM7 kinase, SMAD2, as well as C-terminally truncated SMAD2 revealed that TRPM7 phosphorylates SMAD2 in a dose dependent manner but fails to phosphorylate the truncated SMAD2. Thus, the C-terminal Ser465/467-motif of SMAD2 was identified as a novel substrate for the TRPM7 kinase [20]. The upregulation of the integrin αE, also known as CD103, enables T cells to migrate into the gut epithelium [86,87] and is dependent on SMAD2 TGF-β/SMAD2 signalling cascades [88], which was significantly impaired in TGF-β-treated *Trpm7^R/R^* T cells. Using a chromatin immunoprecipitation (ChIP) assay, the authors demonstrated a defective binding of SMAD2 to the *Itgae* (CD103) promoter in *Trpm7^R/R^* T cells in response to TGF-β. Consequently, the expression of the gene encoding for CD103, *Itgae*, was also significantly reduced in primary *Trpm7^R/R^* IELs as well as in response to TGF-β and T cell receptor co-stimulation in naïve *Trpm7^R/R^* T cells. Consistently, the expression of the signature transcription factor for T_H_17 cells, *Rorc*, as well as the cytokine IL-17, which both depend on SMAD2 phosphorylation and translocation into the nucleus, were also impaired in *Trpm7^R/R^* T cells (Figure 3). Interestingly, the CD103 expression in *Trpm7^R/R^* CD11c^+^ dendritic cells was normal, compared to WT [20]. If or how TRPM7 kinase is triggered via TGF-β stimulation in T cells is still under investigation. One emerging concept, however, could involve a constitutively active TRPM7 kinase that phosphorylates SMAD2 once it is anchored to the plasma membrane following TGF-β receptor activation (Figure 3). Importantly, this selective defect of SMAD2 signalling in T cells culminating in reduced pro-inflammatory T_H_17 cell differentiation, while leaving anti-inflammatory T_reg_ cell differentiation unaffected (Figure 4A,B), highlights the essential function of TRPM7 kinase in immune homeostasis [20]. These results suggest that TRPM7 kinase might serve as molecular switch from pro-inflammatory to anti-inflammatory milieu and highlights the potential of TRPM7 kinase inhibition for the treatment of pro-inflammatory diseases.

To date, very little is known regarding the role of TRPM7 in human lymphocytes. Pharmacological inhibition of TRPM7 in a human T cell line results in growth arrest and reduced proliferation [89]. TRPM7 was suggested to be involved in the migration of activated human T cells, where it is located in the uropod, in conjunction with the calcium-activated potassium channel, K_Ca_3.1, facilitating T cell migration. Knock-down via siRNA resulted in a significant reduction in number and velocity of migrating cells [90]. Moreover, TRPM7 was associated with TNF-α-induced necroptosis in T cells. Knock-down of TRPM7 in a T cell line protected it from necroptosis [91]. Nonetheless, it will be necessary to determine if the observed crucial functions of TRPM7 kinase and channel moieties in murine lymphocytes also applies to human counterparts. 

## 3. TRPM7-Mediated Hematologic and Inflammatory Diseases

### 3.1. Hypomagnesaemia and TRPM7 Kinase in Delayed-Type Hypersensitivity Reactions

Mg^2+^ is a vital mineral macronutrient. Considering that low serum Mg^2+^ levels have also been linked to memory decline, neurodegenerative diseases, decrease in muscle performance, heart failure, certain cancers, autoimmune diseases and allergic reactions, it is of critical importance to further identify the mechanisms regulating the availability of this macronutrient [92,93]. Recently, it was shown that TRPM7, in conjunction with its sister channel TRPM6, regulates systemic Mg^2+^ homeostasis via absorption of Mg^2+^ in the intestine [94]. The interconnection between nutrient metabolism and the immune system occurs at many levels, ranging from endocrine signalling to direct sensing of nutrients by immune cells [95]. Interestingly, adult *Trpm6*-deficient mice suffered from hypomagnesaemia and displayed a degeneration of many lymphoid organs. The thymus of mutant mice was only rudimentary present with an essentially undistinguishable cortex region. Also, the red pulp of the spleens of *Trpm6*-deficient mice was substantially reduced. Dietary Mg^2+^ supplementation rescued these phenotypes, indicating that indeed hypomagnesaemia was responsible for the observed deficits [94].

While homozygous genetic deletion of the ubiquitously expressed TRPM7 kinase domain in mice leads to early embryonic lethality, heterozygous *Trpm7^+/Δk^* mice are viable and develop a severe hypomagnesaemia upon Mg^2+^ restriction, leading to increased mortality, susceptibility to seizures as well as prevalence for allergic hypersensitivity [4]. It is known that low, systemic Mg^2+^ levels correlate with cell-extrinsic enhancement of systemic inflammatory and allergic responses [96]. To evaluate the level of delayed-type hypersensitivity responses in *Trpm7^+/Δk^* mice, oxazolone sensitization experiments were performed. *Trpm7^+/Δk^* mice displayed an elevated oxazolone-induced contact hypersensitivity, compared to WT [4]. Interestingly, homozygous mice with genetic inactivation of TRPM7 kinase activity, via a point mutation within the active site of the kinase, *Trpm7^R/R^*, were viable and did not develop hypomagnesaemia or hypersensitivity responses. In fact, they even displayed reduced oxazolone-induced delayed type hypersensitivity responses [30]. Their systemic Mg^2+^ and Ca^2+^ levels were similar to WT, as the channel function was not affected by the point mutation [29,30,32]. Since allergic reactions are triggered by mast cell-mediated histamine release, the role of TRPM7 in mast cell degranulation and histamine release was studied using *Trpm7^+/+^*, *Trpm7^+/Δk^* and *Trpm7^R/R^* mice. As reported, degranulation and histamine release proceeded independently of TRPM7 channel function. However, as extracellular Mg^2+^ was essential to control unperturbed IgE-DNP-dependent exocytosis and removal of Mg^2+^ exaggerated histamine release, the observed differences in hypersensitivity responses could be attributed to the different systemic Mg^2+^ levels in *Trpm7^+/Δk^* versus *Trpm7^R/R^* mice. G-protein-coupled receptor stimulation revealed strong suppression of histamine release in both kinase-deficient mast cells (*Trpm7^+/Δk^* and *Trpm7^R/R^*), whereas removal of extracellular Mg^2+^ caused the phenotype to revert, suggesting that the TRPM7 kinase activity regulates murine mast cell degranulation by changing its sensitivity to intracellular Ca^2+^ and extracellular Mg^2+^ concentrations [32]. Thus, TRPM7 might inflict its immune-modulatory role by sensing cations via its kinase domain. To date, little is known about activation mechanisms or physiologic substrates of TRPM7 kinase. 

### 3.2. The TRPM7 Channel-Kinase in Arterial Thrombosis and Stroke

TRPM7 kinase has been suggested to regulate myosin IIB filament stability as well as actomyosin contractility by phosphorylating myosin IIA [25]. Recently, it was shown that TRPM7 channel activity also affects myosin IIA activity independently of kinase function. In conditional *Trpm7*-deficient mice (*Trpm7^fl/fl-Pf4Cre^*), TRPM7 modulates platelet function via regulation of cellular Mg^2+^ homeostasis and cytoskeletal myosin IIA activity. Members of a human pedigree with mutations in *TRPM7* (p.C721G), causing disrupted channel activity, suffer from macrothrombocytopenia and arterial fibrosis. The defect in platelet biogenesis is mainly caused by cytoskeletal alterations resulting in impaired pro-platelet formation by *TRPM7*-deficient megakaryocytes, which is rescued by Mg^2+^ supplementation [79]. In contrast, homozygous kinase-dead *TRPM7^R/R^* mice show normal platelet counts, size and morphology, thus suggesting that the lack of TRPM7 channel rather than its kinase activity accounts for the macrothrombocytopenia in *Trpm7^fl/fl-Pf4Cre^* mice [79]. However, the kinase controls platelet function in arterial thrombosis via regulation of Ca^2+^ responses, Syk and PLCγ2 activity. Bone marrow (BM) chimeras revealed that the kinase is not only relevant for platelet function, as both, recipients of WT BM as well as WT recipient of *Trpm7^R/R^* BM, developed reduction in infarct size and improvement of neurological and motoric functions in an in vivo transient middle cerebral artery occlusion (tMCAO) model. Thus, TRPM7 kinase activity in neurons and glial cells may also be critical for the progression of ischemic brain infarction [11]. These findings highlight TRPM7 kinase as a potential target for the treatment of thrombosis thus protecting from stroke or myocardial infarction. 

### 3.3. TRPM7 Kinase Signalling Supports Graft versus Host Reactions

Graft versus host disease (G_V_HD) is the most common side effect of an allogeneic hematopoietic stem cell transplantation (HCST). In this immune reaction the donor T cells recognize the patients human leukocyte antigen (HLA) as foreign, causing an inflammatory cascade [97]. G_V_HD can be divided into acute and chronic, depending on the time of diagnosis [98]. In acute G_V_HD the pre-transplant radiation may cause tissue damage in the host, leading to the activation of antigen presenting cells followed by activation of the donor T cells. This can lead to severe damage of liver, skin, mucosa and the gastrointestinal tract [99]. All in all, G_V_HD causes 15–30% of death after HCST, highlighting the importance of enhancing the understanding of this disease and finding improved treatments. Recently, it was shown that TRPM7 kinase activity promotes gut colonization by T cells in acute G_V_HD. During this process, naïve donor CD4^+^ T cells recognize alloantigens on antigen presenting cells in target organs, including the intestine (Figure 4C). 

The role of different T_H_ subsets and signalling pathways in the pathogenesis of G_V_HD is incompletely understood. To address whether defective intestinal colonization by CD4^+^ T cells lacking TRPM7 kinase activity could affect acute G_V_HD, the bone marrow (BM) of BALB/c WT mice was lethally irradiated and replaced by bone marrow (BM) cells from WT C57BL/6 mice together with WT or *TRPM7^R/R^* splenocytes. As expected, injection of WT splenocytes resulted in massive intestinal damage and most mice died within 35 days after transplantation. In contrast, injection of *TRPM7^R/R^* splenocytes did not cause intestinal damage and resulted in a dramatically increased survival of these mice within the first 60 days after transplantation [20]. These results unravel a fundamental role of TRPM7 kinase in T cell function and suggest a therapeutic potential of kinase inhibitors in averting acute G_V_HD. 

## 4. Conclusions

The involvement of TRPM7 in the pathogenesis of deregulated immune responses highlights the necessity for novel pharmacological tools. TRPM7 represents a new promising drug target for the treatment of pro-inflammatory diseases and hypersensitivity. It is tempting to speculate that pharmacological modulation of TRPM7 may reinstate immune system homeostasis. Particularly appealing is the fact that TRPM7 kinase-deficiency in mice does not result in an obvious phenotype and only moderately affects haemostasis. Thus, TRPM7 kinase inhibition should not cause major side effects. Therefore, new TRPM7 kinase inhibitors and novel kinase substrates have to be identified.
